# Linfoma no hodgkin de células B grandes de la vía biliar simulando un tumor de Klatskin: reporte de un caso

**DOI:** 10.31053/1853.0605.v80.n4.42808

**Published:** 2023-12-26

**Authors:** Clara María Cascio Baccanelli, Andrea Melissa Sian, Pedro Vicente Uad, Jeremias Goransky Patiño

**Affiliations:** 1 Hospital Hospital Italiano de Buenos Aires Argentina; 2 Hospital Italiano San Justo Agustin Rocca. Cirugia Hepatobiliopancreatica Argentina

**Keywords:** cirugía general, linfoma no hodgkin, tumor de klatskin, vía biliar, general surgery, lymphoma, non-hodgkin, klatskin tumor, bile ducts, cirurgia general, linfoma não hodgkin, tumor de klatskin, ductos biliares

## Abstract

La obstrucción de la vía biliar secundaria a un linfoma no hodgkin es extremadamente raro. Es por esto que presentamos el caso de una paciente femenina de 63 años que consulta por ictericia, coluria, acolia y astenia. Un laboratorio presentando un patrón colestásico y una ecografía con la vía biliar intra y extrahepática dilatadas llevaron a realizar una colangioresonancia de abdomen que evidenció una formación expansiva de limites mal definidos que comprometía el conducto hepático común asociado a estenosis del mismo. La sospecha inicial fue un tumor de klatskin y se llevó a cabo la toma de biopsia, cuyo resultado anatomopatológico informó infiltración de linfoma de células B de células grandes doble expresor como tumor primario de la vía biliar. Realizó tratamiento quimioterápico con esquema R CHOP (rituximab, ciclofosfamida, doxorrubicina, vincristina, prednisona) y entró en remisión. Por continuos episodios de colangitis se optó por realizar una hepático yeyuno anastomosis en Y de Roux con reconstrucción de la vía biliar. Actualmente continúa en remisión a 7 años del diagnóstico.

El caso resalta la rareza del linfoma no hodgkin de células B grandes en la vía biliar, y destaca la importancia de la biopsia para un tratamiento eficaz que combina la quimioterapia para la enfermedad de base y la cirugía para las complicaciones obstructivas.

## Abstract (en)

The obstruction of the bile duct secondary to non-Hodgkin lymphoma is extremely rare. That's why we present the case of a 63-year-old female patient who sought medical attention due to jaundice, dark urine, acholia, and weakness. Laboratory results showed a cholestatic pattern, and an ultrasound revealed dilation of the intra and extrahepatic bile ducts, for which a cholangio resonance was ordered. It showed an expansive formation with ill-defined borders compromising the common hepatic duct associated with its stenosis. The initial suspicion was a Klatskin tumor, for which a biopsy was performed, which reported infiltration of a double expressor large B-cell lymphoma as a primary neoplasm of the bile duct. The patient underwent chemotherapy treatment with R-CHOP regimen (rituximab, cyclophosphamide, doxorubicin, vincristine, prednisone) and went into remission. Due to continuous episodes of cholangitis, a Roux-en-Y hepatic jejunal anastomosis with biliary tract reconstruction was performed. Currently, she remains in remission, seven years after the diagnosis.

This case highlights the rarity of large B-cell non-Hodgkin lymphoma in the bile duct and emphasizes the importance of biopsy for effective treatment, combining chemotherapy for the underlying disease and surgery for obstructive complications.

## Resumo

A obstrução da via biliar secundária a um linfoma não Hodgkin é extremamente rara. É por isso que apresentamos o caso de uma paciente do sexo feminino, 63 anos, que procurou assistência médica devido a icterícia, colúria, acolia e astenia. Os resultados laboratoriais indicaram um padrão colestático, e uma ecografia mostrou dilatação da via biliar intra e extra-hepática. Diante disso, foi realizada uma colangiressonância abdominal, que evidenciou uma formação expansiva com limites mal definidos comprometendo o ducto hepático comum, associado à sua estenose. A suspeita inicial foi um tumor de Klatskin, e uma biópsia foi realizada, revelando infiltração de linfoma de células B grandes expressor duplo como o tumor primário no ducto biliar. Ela foi submetida a tratamento quimioterápico com o esquema R-CHOP (rituximabe, ciclofosfamida, doxorrubicina, vincristina, prednisona) e entrou em remissão. Devido a episódios contínuos de colangite, optou-se por realizar uma anastomose hepatico jejunal em Y de Roux com reconstrução da via biliar. Atualmente, ela continua em remissão, sete anos após o diagnóstico.

O caso destaca a raridade do linfoma não Hodgkin de células B grandes na via biliar e ressalta a importância da biópsia para um tratamento eficaz, combinando quimioterapia para a doença subjacente e cirurgia para complicações obstrutivas.

**CONCEPTOS CLAVE**

**Qué se sabe sobre el tema.**

En el mundo son pocos los casos que se reportan de Linfoma no Hodgkin de células B grandes como causa primaria de obstrucción de la vía biliar. Hay reportes de casos similares a este donde la sospecha diagnóstica es confundida inicialmente con un tumor de Klatskin.

**Qué aporta este trabajo.**

Realizar una descripción técnica sobre un abordaje terapéutico combinado (sistémico y quirúrgico) de una patología poco prevalente como etiología de un síndrome altamente incidente en nuestra población.

**Divulgación**

El trabajo relata un reporte de caso sobre una situación clínica singular en la que un paciente presentó síntomas de obstrucción biliar y colestasis, que inicialmente sugirieron la presencia de un tumor de Klatskin, una entidad bien conocida en la patología hepatobiliar. Sin embargo, la posterior evaluación reveló que el origen era un linfoma no Hodgkin de células B grandes primario de la vía biliar. Este diagnóstico inusual implicó la necesidad de un enfoque terapéutico adaptado a la naturaleza del linfoma. El reporte detalla la presentación clínica, los métodos diagnósticos y el tratamiento implementado para abordar esta específica manifestación de linfoma, subrayando la importancia de considerar diagnósticos diferenciales.

## Introducción

La obstrucción de la vía biliar de causa neoplásica puede desarrollarse como consecuencia de diversas patologías. Dentro de ellas, las más frecuentes son tumores de páncreas y tumores de la vía biliar, incluyendo el tumor de klatskin, siendo una de las más infrecuentes la compresión extrínseca por síndromes linfoproliferativos. El linfoma no hodgkin representa el 1-2% de las causas de obstrucción maligna de la vía biliar ^1^ .

Si bien los síndromes linfoproliferativos como el linfoma no hodgkin son muy poco frecuentes, pueden a través de un crecimiento descontrolado comprimir la vía biliar y generar un síndrome coledociano. Los linfomas no Hodgkin (LNH) son un grupo heterogéneo de trastornos linfoproliferativos que se originan en los linfocitos B, T o natural killer (NK). La literatura sugiere que dentro de este grupo de linfoma no hodgkin, se afectan en un 90% de los casos las células B, en un 10% las células T, dejando así una proporción muy pequeña menor al 1% de casos de linfomas de células NK ^2^ .

Si bien hay casos descritos en la literatura sobre síndromes linfoproliferativos como causa de síndromes coledocianos, el linfoma de células B grandes específico no es algo que esté informado como causante habitual de este cuadro.

En este reporte de caso, exponemos el caso de una paciente con una presentación atípica de obstrucción de la vía biliar causado por un linfoma no hodgkin de células B grandes como tumor primario que simuló inicialmente ser un klatskin. A su vez realizaremos una revisión de la literatura existente hasta el momento.

## Materiales y Métodos

Artículo de tipo caso clínico. Se obtuvieron los datos de manera retrospectiva a partir de la historia clínica de la paciente. Se complementó el análisis a partir de la búsqueda bibliográfica. Se respetaron los principios de la Declaración de Helsinki.

## Presentación del caso clínicos

Presentamos el caso de una paciente femenina de 63 años que consulta a la guardia por ictericia asociada a coluria, acolia y astenia de 4 días de evolución (año 2016). Al examen físico la paciente se encontraba estable hemodinámicamente, con ictericia leve evidenciada en piel y mucosas, y un abdomen blando, depresible e indoloro a la palpación. Al interrogatorio la paciente negó factores de riesgo para enfermedades infectocontagiosas. Se solicitó un laboratorio que evidenció un patrón colestásico: bilirrubina total de 7 mg/dL, una bilirrubina directa de 5.5 mg/dL, una fosfatasa alcalina de 408 UI/L, una transaminasa glutamico oxalacetica (GOT) de 166 UI/L, una transaminasa glutamico piruvica (GPT) de 271 UI/L, sin leucocitosis ni anemia asociada. La ecografía informó vía biliar intra y extrahepáticas dilatadas (13mm) con presencia de material hipoecoico en tercio medio, no logrando visualizar su porción distal.

Se solicitó una colangioresonancia magnética nuclear de abdomen con contraste cuyo informe evidenció una formación expansiva de límites mal definidos que comprometía el conducto hepático común (CHC) y se extendía en sentido caudal (Figura 1).

**Figura N°1.**
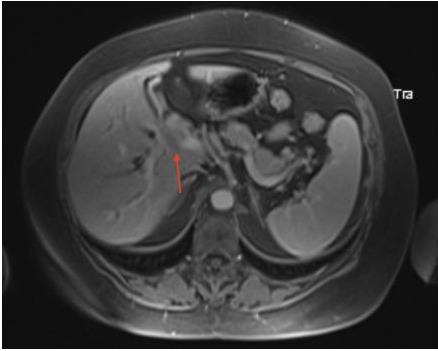
Formación expansiva de límites mal definidos que compromete el conducto hepático común (CHC) y se extiende en sentido caudal.

Se identificó una estenosis en el CHC que se extendía aproximadamente 17 mm generando disociación de los conductos biliares intrahepáticos, con tercio medio e inferior del colédoco con adecuado calibre (Figura 2). Dicho proceso englobaba la vena porta y comprometía la arteria hepática en su trayecto a través del hilio, en asociación con adenomegalias regionales.

**Figura N°2.**
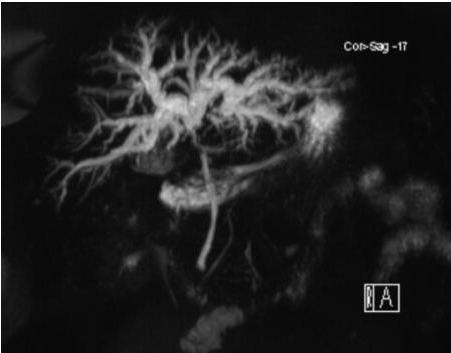
Estenosis en el CHC que se extiende aproximadamente 17 mm. generando disociación de los conductos biliares intrahepáticos, con tercio medio e inferior del colédoco de adecuado calibre

Por la ubicación y características del proceso neoformativo, se sospechó de un tumor de Klatskin y se solicitaron marcadores tumorales para evaluar el mismo. Ante el aumento de los valores de bilirrubina y de la negatividad de los marcadores tumorales alfafetoproteína, Ca 19.9 y el antígeno carcinoembrionario (CEA), se decidió su internación para una posible resección quirúrgica.

Se realizó una cirugía convencional, donde se observó compromiso biliar asociado a compromiso vascular bilateral de la arteria hepática y la vena porta por lo cual se decidió la irresecabilidad del mismo. Se tomaron biopsias de la masa del pedículo hepático para una posterior quimioterapia. La paciente evolucionó adecuadamente, sin complicaciones postoperatorias.

El resultado de anatomía patológica informó infiltración por linfoma B de células grandes doble expresor. La paciente se encontraba clínicamente estable y en condiciones de comenzar con el tratamiento quimioterápico por lo que se decide comenzar con un esquema RCHOP (rituximab, ciclofosfamida, doxorrubicina, vincristina, prednisona). Cumplió 5 ciclos y por ser doble expresor complemento con un sexto ciclo con dosis ascendente EPOCH (etopósido, prednisona, vincristina, ciclofosfamida y doxorrubicina). Evolucionó favorablemente con el esquema electo, presentando solo dos internaciones entre los diferentes ciclos, la primera por neutropenia febril secundario a una mucositis, y la segunda por una colangitis que resolvió con antibióticos sin necesidad de extraer el stent.

Se realizó una tomografía por emisión de positrones (PET) previo al cuarto ciclo del esquema quimioterápico donde se evidencio una disminución del tamaño de la lesión hepática conocida en el segmento III a nivel del carrefour la cual media 12.5 mm, previo de 25 mm (Figura 3A).

La paciente finalizó el esquema quimioterápico a comienzos del 2017 y entró en remisión pero comenzó con reiteradas intercurrencias (Figura 3B). Presentó numerosos episodios de colangitis asociados a la presencia de una estenosis del CHC, por lo cual se realizaron reiteradas colangiopancreatografías retrógradas endoscópicas (CPRE) para el drenaje de la vía biliar con múltiples recambios de stents biliares. La estenosis generó la sospecha de una recaída, por lo cual se decidió realizar una colangioscopia con spyglass para la toma de biopsias bajo visión directa. El resultado fue negativo para células atípicas, confirmando que la causa de la estenosis fue una fibrosis como respuesta del linfoma a la quimioterapia.


**Figura N°3A.**
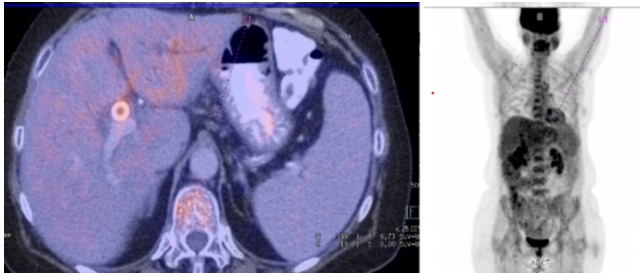
PET que evidencia disminución del tamaño de la lesión hepática conocida en el segmento III a nivel del carrefour la cual medía 12.5 mm, previo de 25 mm. en comparación a última RMN.

**Figura N°3B.**
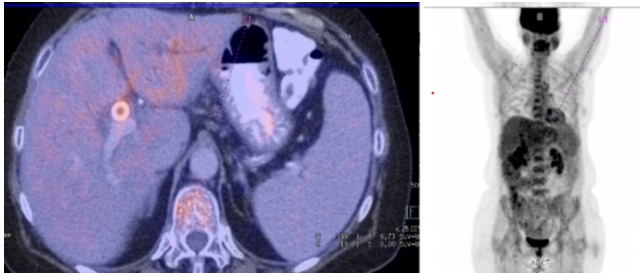
PET en remisión.


Debido a la gran cantidad de intercurrencias en la paciente desde su remisión (año 2017), se decidió en un ateneo multidisciplinario, una resolución quirúrgica (año 2020). Se llevó a cabo una cirugía programada, donde se realizó una **hepático yeyuno anastomosis** **en Y de Roux y una colecistectomía** (Figura 4). Requirió inotrópicos a dosis bajas y antibiótico empírico con imipenem pero evolucionó favorablemente en la terapia intensiva. Se otorgó el alta al séptimo día sin complicaciones.

**Figura N° 4.**
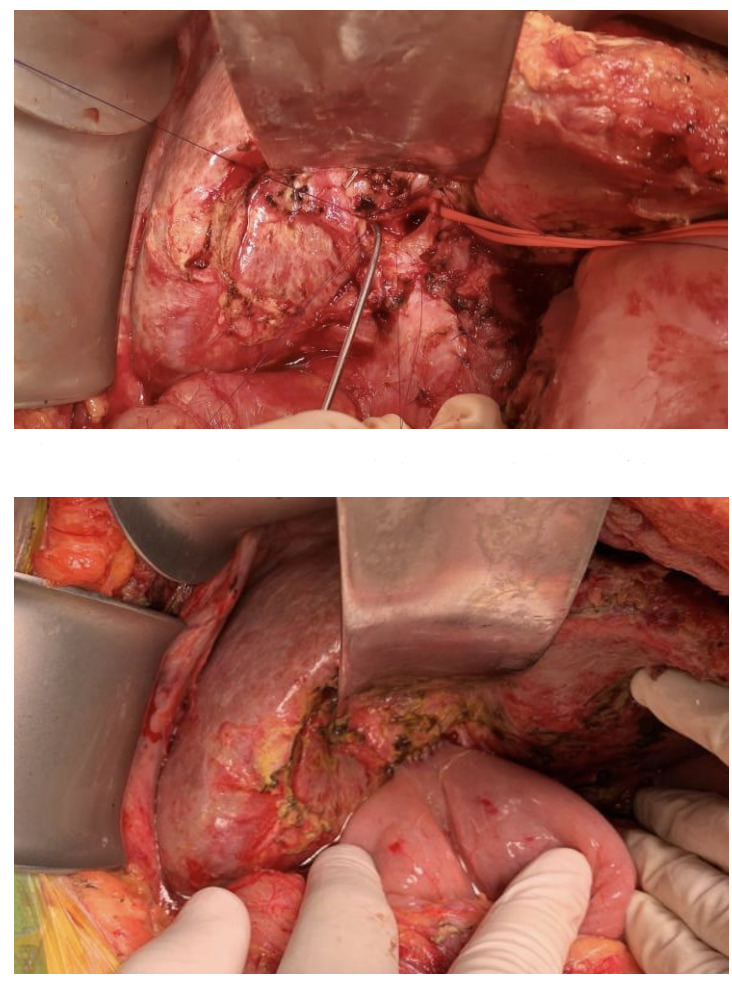
Yeyuno anastomosis en Y de Roux con reconstrucción de la vía biliar.

Se realizó un seguimiento ambulatorio con estudios de resonancia magnética y laboratorios, los cuales no evidencian recidiva de la enfermedad a 6 años entrar en remisión (Figura 5).

**Figura N° 5.**
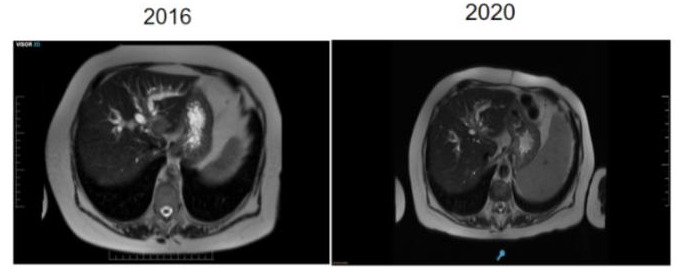
Seguimiento y control con RMN. Al 2020 no hay evidencia de linfoma.

## Discusión

Dentro de los linfomas no hodgkin los linfomas difusos de células B grandes suelen ser los más frecuentes. La incidencia aumenta en hombres y a medida que avanza la edad ^2^ . Si bien es habitual presentar síntomas B (sudoración nocturna, pérdida de apetito, pérdida de peso, etc.) nuestra paciente los negaba, por lo que la primera sospecha diagnóstica no fue un linfoma.

A pesar de que se deben considerar las causas neoplásicas dentro de los síndromes coledocianos, estas no son la primera opción diagnóstica a considerar.

Mucho más infrecuente es el compromiso biliar secundario a patología linfoproliferativa. Los estudios de diagnósticos por imágenes, ya sea tomografía o colangioresonancia, pueden orientar a un diagnóstico etiológico. Si bien son estudios de alta definición, muchas veces, al igual que en el caso de nuestra paciente, la única forma de llegar a un diagnóstico definitivo es un estudio anatomopatológico después de la cirugía. La toma de la biopsia se puede llevar a cabo a través de CRPE o cirugía convencional. Si se logra realizar un correcto diagnóstico tumoral endoscópico, iniciar con quimioterapia es un tratamiento acertado ya que ayuda a disminuir el tamaño o erradicar el tumor. En la mayoría de los reportes de casos leídos el tratamiento quimioterápico elegido para tratar el linfoma de células B grandes de vía biliar fue el R-CHOP como el que se le brindó a nuestra paciente ^1, 3-5^ . La cirugía idealmente se reserva para complicaciones obstructivas y para cuando la quimioterapia fracasa ^3^ . La combinación de ambos tratamientos resultó exitoso logrando la remisión de la enfermedad de base y sus consecuencias biliares.

## Conclusión

Este reporte de caso destaca la singularidad de un linfoma no hodgkin de células B grandes como tumor primario de la vía biliar que simuló un tumor de Klatskin. La presentación atípica de este linfoma no generó sospechas diagnósticas con la clínica y los estudios complementarios, por lo cual fue indispensable una biopsia para orientar el diagnóstico definitivo y el tratamiento.

Este caso contribuye a la literatura y la educación médica ya que enfatiza sobre una patología sumamente infrecuente del enorme abanico diagnóstico que incluyen las patologías neoplásicas de la vía biliar
